# Trends in disparities in COVID hospitalizations among community‐dwelling residents of two counties in Connecticut, before and after vaccine introduction, March 2020–September 2021

**DOI:** 10.1111/irv.13082

**Published:** 2022-12-12

**Authors:** Caroline McWilliams, Laura Bothwell, Kimberly Yousey‐Hindes, James L. Hadler

**Affiliations:** ^1^ Epidemiology of Microbial Diseases Yale School of Public Health New Haven Connecticut USA; ^2^ Connecticut Emerging Infections Program Yale School of Public Health New Haven Connecticut USA

**Keywords:** census, COVID‐19 hospitalization, COVID‐19 vaccination, racial/ethnic disparities, socioeconomic status

## Abstract

**Background:**

Prior to the introduction of vaccines, COVID‐19 hospitalizations of non‐institutionalized persons in Connecticut disproportionately affected communities of color and individuals of low socioeconomic status (SES). Whether the magnitude of these disparities changed 7–9 months after vaccine rollout during the Delta wave is not well documented.

**Methods:**

All initially hospitalized patients with laboratory‐confirmed COVID‐19 during July–September 2021 were obtained from the Connecticut COVID‐19‐Associated Hospitalization Surveillance Network database, including patients' geocoded residential addresses. Census tract measures of poverty and crowding were determined by linking geocoded residential addresses to the 2014–2018 American Community Survey. Age‐adjusted incidence and relative rates of COVID‐19 hospitalization were calculated and compared with those from July to December 2020. Vaccination levels by age and race/ethnicity at the beginning and end of the study period were obtained from Connecticut's COVID vaccine registry, and age‐adjusted average values were determined.

**Results:**

There were 708 COVID‐19 hospitalizations among community residents of the two counties, July–September 2021. Age‐adjusted incidence was the highest among non‐Hispanic Blacks and Hispanic/Latinx compared with non‐Hispanic Whites (RR 4.10 [95% CI 3.41–4.94] and 3.47 [95% CI 2.89–4.16]). Although RR decreased significantly among Hispanic/Latinx and among the lowest SES groups, it increased among non‐Hispanic Blacks (from RR 3.2 [95% CI 2.83–3.32] to RR 4.10). Average age‐adjusted vaccination rates among those ≥12 years were the lowest among non‐Hispanic Blacks compared with Hispanic/Latinx and non‐Hispanic Whites (50.6% vs. 64.7% and 66.6%).

**Conclusions:**

Although racial/ethnic and SES disparities in COVID‐19 hospitalization have mostly decreased over time, disparities among non‐Hispanic Blacks increased, possibly due to differences in vaccination rates.

## 1 INTRODUCTION

Coronavirus disease, or COVID‐19, is an infectious disease caused by the SARS‐CoV‐2 virus. Although many people infected with SARS‐CoV‐2 are asymptomatic or experience mild to moderate respiratory symptoms, others become seriously ill and require hospitalization. Individuals at any age can become seriously ill and die from COVID‐19; however, older individuals and people with underlying health conditions such as cancer, cardiovascular disease, and diabetes are at greater risk for hospitalization and death from COVID‐19.^1^ Hospitalization is an important indicator of COVID‐19 severity and more accurately reflects which demographic groups within any age group are at higher risk of getting infected with SARS‐CoV‐2 than viral testing, which is prone to testing biases.^2^

Early in the pandemic, it became clear that people of color in the United States were being infected, requiring hospitalization, and dying of COVID‐19 at higher rates than White individuals. Coronavirus exposed and amplified existing racial/ethnic and socioeconomic health disparities in the United States. Although describing racial and ethnic COVID‐19 disparities is important, to aid in interpretation and help minimize the risk of promoting racial stereotypes, socioeconomic data should be collected and reported alongside racial and ethnic data.^3^, ^4^

Prior to this study, two previous studies were conducted using the COVID‐19‐Associated Hospitalization Surveillance Network (COVID‐NET) data in Connecticut. The first study explored racial/ethnic and socioeconomic status (SES) disparities in COVID‐19 hospitalizations during the first “lockdown” wave of the pandemic (March–May 2020) in New Haven and Middlesex Counties.^5^ The second study was conducted during the first half of the second wave of the pandemic, prior to vaccine availability (July–December 2020).^6^ Both studies found significant racial and ethnic disparities in COVID‐19 hospitalizations.^5^, ^6^

During the “lockdown” wave, higher incidence of hospitalization was associated with increasing age, increasing levels of census tract poverty and crowding, and non‐Hispanic Black and Hispanic/Latinx race/ethnicity.^5^ Furthermore, the study found that non‐Hispanic Black race and Hispanic/Latinx ethnicity were more strongly and independently associated with incidence of hospitalization regardless of poverty or crowding status.^5^ Previous studies of influenza hospitalization data in New Haven County had found that census tract poverty and crowding levels were more strongly associated with hospitalization than race/ethnicity.^7^, ^8^ The researchers hypothesized that the epidemiology of SARS‐CoV‐2 hospitalization was greatly impacted by the “stay at home” orders, which protected the majority of the population from exposure, with the exception of “essential workers” and their household contacts.^5^ Essential workers with decreased access to personal protective equipment and social support disproportionately included people of color, accounting for the racial/ethnic disparities in hospitalization incidence.^5^

Hospitalization data from July to December 2020 also revealed that race and ethnicity were more strongly associated with higher incidence of hospitalization compared with census tract levels of poverty and crowding.^6^ However, the racial/ethnic disparities (as well as SES disparities) in hospitalization were smaller from July to December 2020 than those found during the initial “lockdown” time period. The researchers attributed this reduction to increased accessibility of personal protective equipment and the lifting of restrictions on non‐essential businesses, gatherings, and activities outside the home which led to exposures across a wider diversity of ages, SES, and racial/ethnic groups.^6^

**FIGURE 1:**
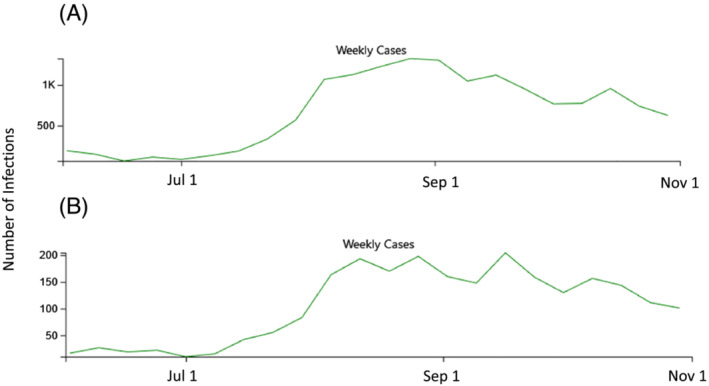
(A and B) Number of laboratory‐confirmed SARS‐CoV‐2 positive persons diagnosed in New Haven (A) and Middlesex Counties (B), Connecticut, June–October 2021. *Source*: https://covid.cdc.gov/covid‐data‐tracker/#county‐view?list_select_state=Connecticut&data‐type=CommunityLevels

**FIGURE 2:**
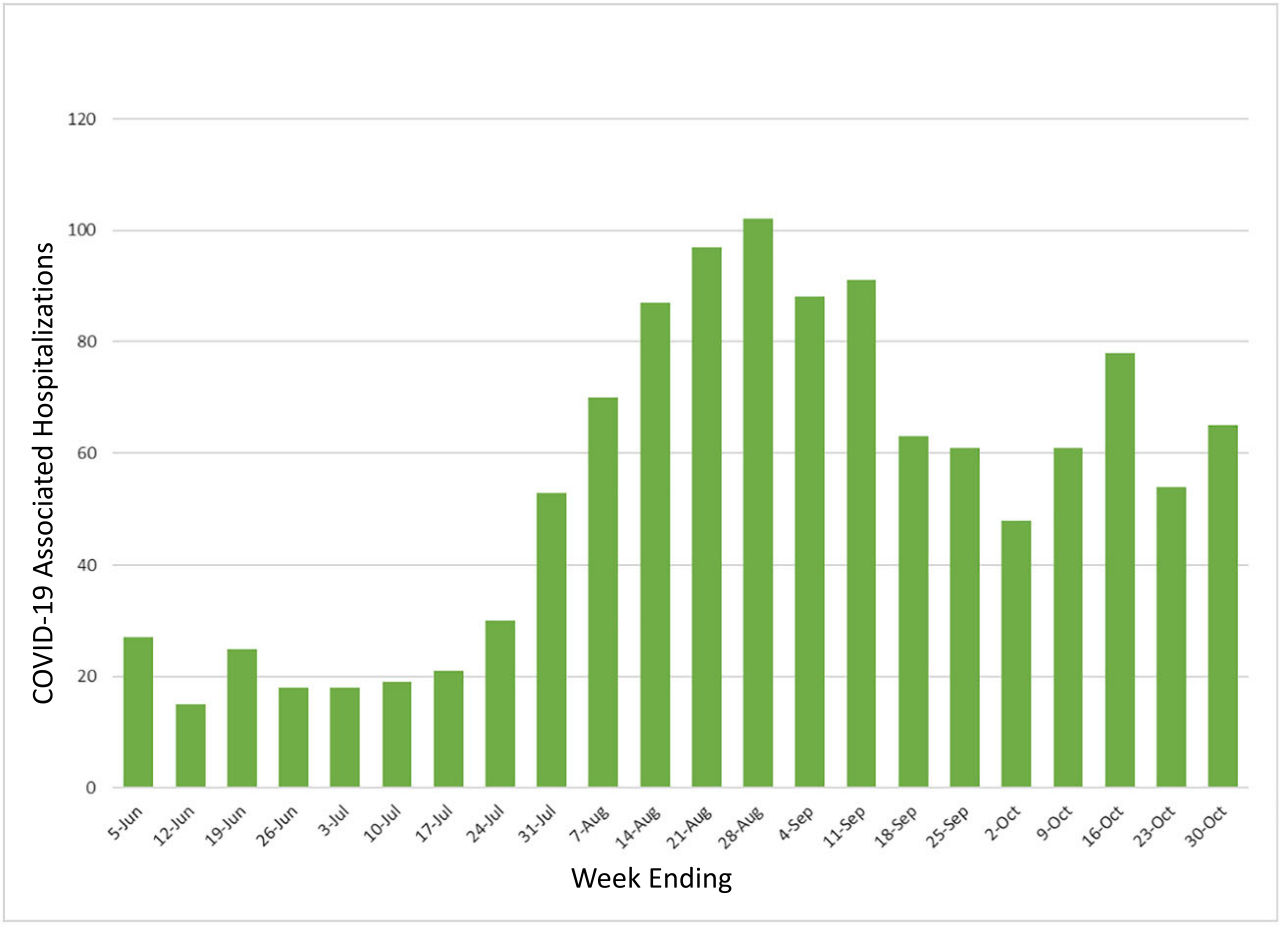
Number of persons hospitalized with laboratory‐confirmed SARS‐CoV‐2 in New Haven and Middlesex Counties, Connecticut, June–October 2021. *Source*: Connecticut Emerging Infections Program, COVID‐NET Surveillance

The first objective of this study was to determine the population‐based epidemiology of COVID‐19 hospitalization in July–September 2021 in the same study areas, Middlesex and New Haven counties, during the Delta variant wave and beginning after all persons 12 years and older had the opportunity to be vaccinated against COVID‐19 for at least 2–7 months.^9^, ^10^, ^11^ The second was to determine trends in racial/ethnic and SES hospitalization incidence disparities by comparing these results with the two previous pre‐vaccination time periods. By September 30, 2021, an estimated 75.5% of Connecticut's population age ≥ 12 had received two vaccination doses and 81.6% had received at least one dose.^12^, ^13^ For context, Figures 1 and 2 show the epidemic curves of infection and hospitalization from June to October 2021 in the two counties included in the analysis.

## 2 METHODS

### 2.1 Surveillance data

This study used the COVID‐NET data gathered by the Connecticut Emerging Infections Program. Cases of initial (during the study time period) hospitalizations of residents of New Haven and Middlesex Counties with laboratory‐confirmed COVID‐19 during July–September 2021 were identified through hospital, laboratory, and admission databases as well as infection control logs. Demographic information including age, race/ethnicity, gender, and residential address were collected for each case and confirmed via electronic medical records. Case home addresses were geocoded and assigned the appropriate census tract. Case home types were recorded as either residential, skilled nursing facility, correctional facility, homeless, hospice, hospitalized since birth, substance use treatment facilities, residential care, group home, or psychiatric facility.

### 2.2 Study population

The selected study population included non‐institutionalized residents of New Haven and Middlesex Counties who were hospitalized for the first time with laboratory‐confirmed COVID‐19 during the time period of July to September 2021. Non‐institutionalized, or community‐dwelling individuals, were defined as people living at a residential address at the time of their hospitalization, which excluded people admitted to the hospital from a skilled nursing facility, correctional facility, rehabilitation facility, homeless shelter, and so on. The population included individuals of any age.

### 2.3 Census data

We used the 2010 census tracts and the American Community Survey (ACS) 5‐year estimates of poverty and crowding from the years 2014–2018. These were the same data sources that had been used by the two studies conducted earlier in the pandemic so that we could compare our results; census data were identified and downloaded online (censusdata.gov). The ACS data for poverty levels gave the percent of individuals in each census tract in New Haven and Middlesex Counties who were living in poverty. The ACS data for crowding gave the number of households where the ratio of people per room was greater than one for each census tract in New Haven and Middlesex Counties. We then divided this number by the total number of people living in each individual census tract to determine the prevalence of crowding. Poverty and crowding data were categorized into four groups. Level of poverty was determined by percent of residents living below the federal poverty line: 0–4.9% was categorized as *very low levels of poverty*, 5–9.9% as *low levels of poverty*, 10–20% as *moderate levels of poverty*, and 20% as *high levels of poverty*. Level of crowding was determined by percent of residents living with more than one person per room: 0–0.009% was categorized as *very low levels of crowding*, 0.01–0.25% as *low levels of crowding*, 0.026–0.05% as *moderate levels of crowding*, and 0.05–4% as *high levels of crowding*.

### 2.4 Statistical analysis

Community‐dwelling cases were compared by demographic variables with those excluded because of institutional residence using odds ratios. Crude and age‐adjusted incidence rates by demographic variables were calculated for community‐dwelling cases. Age‐adjusted rates were calculated using the 2000 US standard population proportions.^14^ Chi‐square tests were used to compare hospitalization incidence between demographic and SES groups. Chi‐square for trend was used to determine whether there was a significant trend association between increasing poverty and crowding levels with age‐adjusted incidence. Chi‐square tests and 95% confidence intervals (CI) were used to compare the case characteristics of community‐dwelling patients hospitalized due to COVID‐19 for this study and the study conducted from July to December 2021. Average age‐adjusted rates of vaccination by race/ethnicity were calculated using age and race/ethnicity‐specific data made available online from the Connecticut Department of Public Health using data from the weeks ending July 7 and September 29, 2021, and averaging the results.^13^ All statistical analyses were performed using SAS version 9.4 and Epi Info.

## 3 RESULTS

There were 751 cases of initial hospitalization with laboratory‐confirmed SARS CoV‐2 during July–September 2021. Of these 751 cases, 708 were hospitalized from a residential address and 43 from an institution, with the majority of the latter from long‐term care facilities.

For age‐adjusted relative risks of hospitalized cases in community‐dwelling individuals, non‐Hispanic Black individuals were 4.10 times more likely and Hispanic/Latinx individuals 3.47 times more likely to be hospitalized than White individuals (Table 1). Relative risk also increased with age, with those 50–64 years of age 1.66 times more likely and those older than 85 years 4.58 times more likely than 18‐ to 49‐year olds to be hospitalized with COVID‐19. Individuals living in census tracts with high levels of poverty were 2.31 times more likely to be hospitalized than individuals living in census tracts with very low levels of poverty. Similarly, individuals living in census tracts with high levels of crowding were 2.02 times more likely to be hospitalized than individuals living in very low levels of crowding.

**TABLE 1:** Number and age‐adjusted incidence by demographic variables of community‐dwelling individuals hospitalized with COVID in New Haven and Middlesex Counties, CT, July–September 2021

	*N*	Total population	Adj RR	95% CI
Total hospitalized patients	708	1,028,153	—	—
Gender				
Male	383 (54.1)	495,998	1.38	1.18–1.61
Female	325 (45.9)	532,155	Ref	Ref
Race				
NH White	381 (53.8)	725,528	Ref	—
NH Black	166 (23.4)	109,019	4.10	3.41–4.94
Hispanic/Latinx	123 (17.4)	137,577	3.47	2.89–4.16
Asian/Pacific Islander	10 (1.4)	34,140	1.24	0.76–2.02
Other	28 (4.0)	21,889	—	—
Age group				
0–17	10 (1.4)	228,072	0.08	0.04–0.16
18–49	235 (33.2)	441,329	Ref	—
50–64	185 (26.1)	209,159	1.66	1.37–2.01
65–74	119 (16.8)	74,130	3.01	2.42–3.76
75–84	95 (13.4)	49,238	3.62	2.86–4.60
85+	64 (9.0)	26,225	4.58	3.48–6.04
Month of hospitalization				
July	119	1,028,153	—	—
August	344	1,028,153	—	—
September	245	1,028,153	—	—
Community poverty levels				
Very low	191 (27.4)	366,844	Ref	—
Low	156 (22.4)	270,104	1.10	0.87–1.38
Moderate	163 (23.4)	197,588	1.78	1.42–2.21
High	186 (26.7)	193,617	2.31	1.87–2.84
Community crowding levels				
Very low	269 (38.6)	508,471	Ref	—
Low	208 (29.9)	274,286	1.51	1.24–1.83
Moderate	133 (19.1)	144,042	1.94	1.57–2.41
High	86 (12.4)	101,354	2.02	1.58–2.57

Abbreviations: CI, confidence intervals; NH, non‐Hispanic.

However, when race/ethnicity was stratified by census tract poverty and crowding levels, there remained stark differences in the age‐adjusted relative risks of non‐Hispanic Black and Hispanic/Latinx individuals compared with White individuals in each level of poverty and crowding (Table 2). Non‐Hispanic Black and Hispanic/Latinx individuals were 2.3 to 6.0 times more likely to be hospitalized with COVID‐19 than White individuals living in census tracts with the equivalent levels of poverty and crowding. As the pandemic continued in Connecticut, a progressively smaller percentage of COVID‐19 hospitalizations occurred among institutionalized populations, from 30.4% during the “lockdown” period to 14.6% in July–December 2020 and to 5.7% in July–September 2021 (Table 3). Additionally, there was a significant increase in the percentage of all hospitalizations for individuals aged 18–49 years old during July–September 2021 compared with July–December 2020. Furthermore, there was a statistically significant increase in the incidence of hospitalization for non‐Hispanic Black individuals compared with July–December 2020. The relative risk of non‐Hispanic Black persons compared with White persons increased from 3.20 to 4.10, whereas the relative risk of Hispanic/Latinx individuals compared with White persons decreased from 4.73 to 3.47 (Table 4). However, the high versus very low relative risks in both levels of poverty and crowding remained relatively stable between the July–December 2021 and July–September 2020 time periods.

**TABLE 2:** Age‐adjusted and relative age‐adjusted incidence of laboratory‐confirmed SARS‐CoV‐2 hospitalizations for community‐dwelling individuals in New Haven and Middlesex Counties, CT, July ‐ September 2021, by race/ethnicity stratified by census tract poverty and crowding levels

	Adj IR/100,000	Adj RR	95% CI
Census tract levels of poverty			
Very low			
NH White	37.4	—	—
NH Black	141.9	3.9	(2.16–7.08)
Hispanic/Latinx	141.6	3.8	(2.46–6.00)
Low			
NH White	36.6	—	—
NH Black	166.1	4.4	(2.79–7.06)
Hispanic/Latinx	149.1	4.0	(2.65–6.07)
Moderate			
NH White	50.8	—	—
NH Black	151.4	3.0	(2.05–4.40)
Hispanic/Latinx	136.7	2.7	(1.85–3.96)
High			
NH White	42.9	—	—
NH Black	169.2	4.0	(2.59–6.18)
Hispanic/Latinx	135.4	3.2	(2.08–4.99)

Census tract level of crowding			
Very low			
NH White	36.6	—	—
NH Black	129.8	3.5	(2.41–5.17)
Hispanic/Latinx	89.6	2.4	(1.68–3.56)
Low			
NH White	39.8	—	—
NH Black	233.6	6.0	(4.30–8.24)
Hispanic/Latinx	203.2	5.1	(3.70–7.17)
Moderate			
NH White	47.5	—	—
NH Black	143.1	3.0	(1.96–4.74)
Hispanic/Latinx	147.5	3.1	(2.05–4.83)
High			
NH White	57.8	—	—
NH Black	131.5	2.3	(1.30–3.92)
Hispanic/Latinx	130.8	2.3	(1.36–3.82)

Abbreviations: CI, confidence intervals; IR, incidence rate; NH, non‐Hispanic.

**TABLE 3:** Comparison of community‐dwelling patients hospitalized for COVID‐19 from March–June 30, 2020, July–December 31, 2020, and July 1–September 30, 2022, residing in New Haven and Middlesex Counties, CT

Case characteristics	Mar–Jun 2020	Jul–Dec 2020	Jul–Sep 2021
	*N* cases (%)	*N* cases (%)	*N* cases (%)
Total hospitalized cases	2733	6967	751
Institutionalized cases	832 (30.4)	1018 (14.6)	43 (5.7)^***^
Total community COVID‐NET cases	1901 (69.6)	2035 (29.2)	708 (94.3)
Sex			
Male	989 (52.0)	1040 (51.1)	383 (54.1)
Female	912 (48.0)	995 (48.9)	325 (45.9)
Age group			
0–17	16 (0.8)	18 (0.9)	10 (1.4)
18–49	433 (22.8)	406 (22.0)	235 (33.2)^***^
50–64	561 (29.5)	556 (27.3)	185 (26.1)
65+	891 (46.9)	1055 (51.8)	278 (39.3)^***^
Race/ethnicity			
Non‐Hispanic White	778 (40.9)	1148 (56.4)	381 (53.8)
Non‐Hispanic Black	536 (28.2)	350 (17.2)	166 (23.4)^***^
Hispanic/Latinx	419 (22.0)	406 (22.0)	123 (17.4)
Asian/Pacific Islander	33 (1.7)	18 (0.9)	10 (1.4)
Other	—	113 (5.6)	28 (4.0)
Census tract levels of poverty			
Very low	381 (20.0)	567 (27.9)	191 (27.4)
Low	410 (21.6)	513 (25.2)	156 (22.4)
Medium	468 (24.6)	427 (21.0)	163 (23.4)
High	642 (33.8)	528 (26.0)	186 (26.7)
Census tract levels of crowding			
Very low	699 (36.8)	865 (42.5)	269 (38.6)
Low	496 (26.1)	553 (27.2)	208 (29.9)
Medium	359 (18.9)	359 (17.6)	133 (19.1)
High	347 (18.3)	258 (12.7)	86 (12.4)

*Note*: Extended data from that reported in Hadler et al^5^ through June 30, 2020.

^*^ *P*‐value < .05 of the percentage of total July–September 2021 compared with July–December 2020.

^**^ *P*‐value < .01 of the percentage of total July–September 2021 compared with July–December 2020.

^***^ *P*‐value < .001 of the percentage of total July–September 2021 compared with July–December 2020.

**TABLE 4:** Time period comparisons of age‐adjusted relative risks by race/ethnicity and socioeconomic status of residents of New Haven and Middlesex Counties, CT, in March–May 2020, July–December 2020, and July–September 2021

	Mar–Jun 2020	Jul–Dec 2020	Jul–Sep 2021
	aRR (95% CI)	aRR (95% CI)	aRR (95% CI)
Race/ethnicity			
Non‐H Black vs. non‐H White	7.51 (6.71–8.41)	3.20 (2.84–3.60)	4.10 (3.41–4.94)
Hispanic vs. non‐H White	6.57 (5.88–7.35)	4.73 (4.29–5.22)	3.47 (2.89–4.16)
Poverty			
High vs. very low	4.36 (3.82–4.99)	2.48 (2.20–2.81)	2.31 (1.87–2.84)
Med vs. very low	2.68 (2.32–3.10)	1.69 (1.48–1.94)	1.78 (1.42–2.21)
Low vs. very low	1.41 (1.21–1.60)	1.21 (1.06–1.39)	1.10 (0.87–1.38)
Crowding			
High vs. very low	3.40 (2.99–3.87)	2.06 (1.79–2.36)	2.02 (1.58–2.57)
Med vs. very low	2.11 (1.84–2.41)	1.75 (1.53–1.98)	1.94 (1.57–2.41)
Low vs. very low	1.42 (1.25–1.60)	1.27 (1.13–1.43)	1.51 (1.24–1.83)

Abbreviation: CI, confidence intervals.

Vaccination completion rates at both the beginning and end of the study period varied by age group and race/ethnicity (Table 5). Within each race/ethnic group, vaccination rates increased with age. Non‐Hispanic Black persons had the lowest age‐specific vaccination rates in each age group and in the overall age‐adjusted average, which was 14–16% lower than for the non‐Hispanic White and Hispanic/Latinx groups. Although White individuals had higher age‐specific vaccination rates in age groups <45 years, Hispanic/Latinx individuals had higher vaccination rates for age groups 45 and older.

**TABLE 5:** Percent of CT state residents and age‐adjusted average fully vaccinated from July 7 to September 29, 2021, by race/ethic and age groups

	Age group				
Race/ethnic group	12–15 years	16–44 years	45–55 years	55+ years	Age‐adjusted average^a^
Non‐Hispanic Black	18.5–39.2	31.1–43.6	47.2–57.7	64.6–71.8	50.6
Hispanic/Latinx	26.6–51.7	45.7–59.0	65.6–76.2	77.0–83.5	64.7
Non‐Hispanic White	44.0–64.2	57.1–64.4	61.2–67.4	73.9–77.6	66.6

*Note*: Fully vaccinated = two doses of Moderna or Pfizer vaccines or one dose of Johnson and Johnson vaccine. Vaccination rates are underestimates as denominators are full denominators for each group based on the 2020 census, but 5.0–4.5% of vaccinated persons had unknown race/ethnicity.

^a^ Age‐adjusted by the four age groups using 2020 census for CT. Average of age‐adjusted rates for July 7 and September 29, 2021.

## 4 DISCUSSION

One of the most important findings from this study is the persistence of racial/ethnic disparities in COVID‐19 hospitalizations among community‐dwelling individuals in New Haven and Middlesex Counties from July to September 2021. Additionally, race/ethnicity remains more strongly associated with hospitalization than census tract‐based measures of SES.

Inequalities in access to and acceptability of the COVID‐19 vaccine most likely contributed to the disparities, especially in the increased disparity seen in the non‐Hispanic Black population well after the introduction of vaccine. Recent events, such as the water crisis in Flint, high Black maternal mortality rates, and coerced sterilizations in ICE detention centers, provide people of color with many reasons to continue to distrust medical and public health institutions.^15^ Lower rates of self‐reported vaccine acceptability among non‐Hispanic Black and Hispanic/Latinx individuals in the United States reflect this justified mistrust. Compared with non‐Hispanic White individuals, non‐Hispanic Black individuals had 3.84 times the odds of reporting vaccine hesitancy and Hispanic/Latinx individuals had 1.69 times the odds of reporting vaccine hesitancy.^16^ Additionally, disinvestment due to racialized practices and policies has left many communities of color without adequate access to medical care and health‐promoting resources, including vaccines.^15^

Whether due to mistrust, lack of access, or both, at the end of September 2021, Connecticut's vaccine eligible, non‐Hispanic Black population was much less likely to be fully vaccinated than either the Hispanic/Latinx and non‐Hispanic White populations. Notably, the gap in vaccination rates between Connecticut's non‐Hispanic White population and Hispanic/Latinx population is less pronounced than the disparity between the non‐Hispanic White and non‐Hispanic Black populations, and Hispanic/Latinx vaccination rates are higher than the non‐Hispanic White rates among those 45 years and older. Because increased age is associated with a higher likelihood of COVID‐19 hospitalization, the relatively higher rates of vaccination in Hispanic/Latinx persons could explain why the age‐adjusted relative risk for Hispanic/Latinx individuals compared with non‐Hispanic White individuals decreased from 4.73 in the immediate pre‐vaccine era to 3.47 during July–September 2021.

Another important finding from this study is the decrease in the percentage of hospitalized cases that were institutionalized, which may reflect high vaccination rates among persons residing in nursing homes. In early July 2021, approximately 88% of residents in long‐term care facilities located in Connecticut were fully vaccinated; by the end of September, this percentage increased to 92%.^17^

Of note, the increased percentage of hospitalizations among persons 18–49 years old and the decrease in those aged 65 and older observed during this initial vaccine period analysis may reflect relative vaccination rates. In addition, older individuals may also have been more likely to continue avoiding SARS‐CoV‐2 exposure, including wearing masks, due to elevated concern about relative risks of the virus for older populations versus younger populations.

The data suggest that relative vaccination rates can greatly influence COVID hospitalization epidemiology. Targeted efforts are needed to achieve not just high overall vaccination rates but especially among groups with ongoing disparities in severe COVID outcomes, including hospitalization. To achieve higher vaccination rates, it will be important to understand why disparities in vaccination exist and address the fundamental causes.^18^

Limitations of our findings include using vaccination rates for the entire state of Connecticut to explain patterns in New Haven and Middlesex counties and using census tract levels of poverty and crowding to approximate an individual's SES. Further, some of the cases included in our analysis were hospitalized with, not because of, COVID‐19, as all hospitals in the two counties routinely screened all admissions for SARS‐Co‐V infection. We could not eliminate these cases from analysis as, according to COVID‐NET protocol, only approximately 20% of cases had full medical record reviews. If there were differential rates by race/ethnicity of admission for non‐COVID‐19‐related conditions who test positive for SARS‐CoV‐2, then possibly some bias could have been introduced. However, in a sample of 184 cases residing in the catchment area whose hospitalization medical records were reviewed, there were no differences by race/ethnicity in the percentage that were admitted because of COVID‐19.

## 5 CONCLUSION

In summary, the key finding from this analysis is that 2–9 months after the rollout of COVID vaccine to all ages 12 years and older, little progress was achieved since the late pre‐vaccine era in the goal of health equality with respect to hospitalization with COVID‐19. In particular, the relative rate of hospitalization for non‐Hispanic Blacks increased, associated with markedly lower vaccination rates than other racial/ethnic groups. Achieving higher vaccination rates overall, but especially among non‐Hispanic Blacks, demands public health attention and community‐based interventions.

## CONFLICT OF INTEREST

The authors have no conflicts of interest.

## ETHICS STATEMENT

The study was conducted according to the guidelines of the Declaration of Helsinki and approved by the Human Subjects Committee of the Connecticut Department of Public Health (protocol number 958, approved January 18, 2022). Patient consent was waived by the DPH HIC based on responses to each of the criteria for the waiver (45 CFR 46.116(f)(3)).

## AUTHOR CONTRIBUTIONS

**Caroline McWilliams:** Conceptualization; formal analysis; methodology; writing‐original draft. **Laura Bothwell:** Conceptualization; methodology; supervision; writing‐review and editing. **Kimberly Yousey‐Hindes:** Data curation; funding acquisition; resources; writing‐review and editing. **James L Hadler:** Conceptualization; formal analysis; methodology; resources; supervision; writing‐review and editing.

### PEER REVIEW

The peer review history for this article is available at https://publons.com/publon/10.1111/irv.13082.

## Data Availability

Aggregate data on hospitalization rates and selected demographic characteristics of persons hospitalized with COVID‐19 and identified through COVID‐NET are publicly available online (https://gis.cdc.gov/grasp/COVIDNet/COVID19_3.html), although Connecticut‐specific data are not separated from other sites. The dataset for this analysis contains information that could potentially allow for identification of individuals and is not available except through application to the Connecticut Department of Public Health Human Investigations Committee.
